# Une étiologie rare du syndrome de la queue de cheval : l'hémangiome vertébral neuro-agressif

**DOI:** 10.11604/pamj.2017.26.95.8717

**Published:** 2017-02-24

**Authors:** Abderrazzak El Saqui, Mohamed Aggouri

**Affiliations:** 1 Service de Neurochirurgie, CHU Hassan II, Fès, Maroc

**Keywords:** Angiome, agressif, rachis, IRM, chirurgie, Angioma, aggressive, spine, MRI, urgery

## Image en médecine

Un patient âgé de 17 ans, sans antécédents pathologiques notables, admis aux urgences neurochirurgicales pour une fatigabilité progressive à la marche et des fuites urinaires. L'examen clinique a trouvé une discrète diminution de la force musculaire des deux membres inférieurs (paraparésie grade D de Frankel) associée à un discret déficit épicritique sans niveau sensitif. Devant ce tableau de compression de la queue de cheval, une IRM du rachis lombo-sacrée a été réalisée et a montré la présence d'un processus vertébral envahissant la majeure partie du corps vertébral de L3 associée à une épidurite antérieure compressive se prolongeant jusqu'en regard du corps vertébral de L4 (A, B, C). Le patient a été opéré par un abord rétropéritonéal avec une décompression du sac dural. Les suites chirurgicales étaient simples. L'anatomopathologie a confirmé le diagnostic d'angiome capillaire bénin. Apres un recul de 4 ans, le patient a récupéré sa force musculaire des membres inferieur ainsi que ces troubles urinaires. Les hémangiomes vertébraux agressifs représentent une entité très rare. Il s'agit de lésions vasculaires bénignes, souvent uniques, qui se manifestent le plus souvent par des signes de compression médullaire. L'IRM occupe une place importante par le fait qu'elle permet une analyse multiplanaire directe et une caractérisation tissulaire. Elle trouve un intérêt majeur dans le bilan d'extension au niveau épidural et permet une bonne approche du retentissement sur les structures nerveuses.

**Figure 1:**
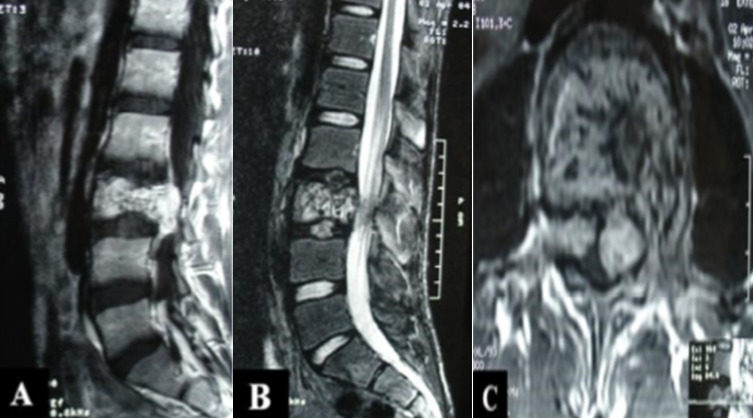
IRM du rachis lombo-sacrée en coupe sagittale, séquence T1 avec Gadolinium (A), séquence T2 (B) et coupe axiale (C), montrant un hémangiome vertébral envahissant la majeure partie du corps vertébral de L3 de même que les deux pédicules, les deux massifs articulaires et l’apophyse transverse gauche

